# Taxonomic characterization of *Pseudomonas hygromyciniae* sp. nov., a novel species discovered from a commercially purchased antibiotic

**DOI:** 10.1128/spectrum.01838-21

**Published:** 2023-09-22

**Authors:** Timothy L. Turner, Sumitra D. Mitra, Travis J. Kochan, Nathan B. Pincus, Marine Lebrun-Corbin, Bettina H. Cheung, Samuel W. Gatesy, Tania Afzal, Sophie H. Nozick, Egon A. Ozer, Alan R. Hauser

**Affiliations:** 1 Department of Microbiology-Immunology, Northwestern University, Feinberg School of Medicine, Chicago, Illinois, USA; 2 Department of Medicine, Northwestern University, Feinberg School of Medicine, Chicago, Illinois, USA; 3 Department of Biology, Northeastern Illinois University, Chicago, Illinois, USA; Emory University School of Medicine, Atlanta, Georgia, USA

**Keywords:** hygromycin B, *Pseudomonas*, microbial ecology, antimicrobial resistance

## Abstract

In an attempt to identify novel bacterial species, microbiologists have examined a wide range of environmental niches. We describe the serendipitous discovery of a novel gram-negative bacterial species from a different type of extreme niche: a purchased vial of antibiotic. The vial of antibiotic hygromycin B was found to be factory contaminated with a bacterial species, which we designate *Pseudomonas hygromyciniae* sp. nov. The proposed novel species belongs to the *P. fluorescens* complex and is most closely related to *P. brenneri*, *P. proteolytica*, and *P. fluorescens*. The type strain *Pseudomonas hygromyciniae* sp. nov. *strain SDM007^T^* (SDM007^T^) harbors a novel 250 kb megaplasmid which confers resistance to hygromycin B and contains numerous other genes predicted to encode replication and conjugation machinery. SDM007^T^ grows in hygromycin concentrations of up to 5 mg/mL but does not use the antibiotic as a carbon or nitrogen source. While unable to grow at 37°C ruling out its ability to infect humans, it grows and survives at temperatures between 4 and 30°C. SDM007^T^ can infect plants, as demonstrated by the lettuce leaf model, and is highly virulent in the *Galleria mellonella* infection model but is unable to infect mammalian A549 cells. These findings indicate that commercially manufactured antibiotics represent another extreme environment that may support the growth of novel bacterial species.

**IMPORTANCE:**

Physical and biological stresses in extreme environments may select for bacteria not found in conventional environments providing researchers with the opportunity to not only discover novel species but to uncover new enzymes, biomolecules, and biochemical pathways. This strategy has been successful in harsh niches such as hot springs, deep ocean trenches, and hypersaline brine pools. Bacteria belonging to the *Pseudomonas* species are often found to survive in these unusual environments, making them relevant to healthcare, food, and manufacturing industries. Their ability to survive in a variety of environments is mainly due to the high genotypic and phenotypic diversity displayed by this genus. In this study, we discovered a novel *Pseudomonas* sp. from a desiccated environment of a sealed antibiotic bottle that was considered sterile. A close genetic relationship with its phylogenetic neighbors reiterated the need to use not just DNA-based tools but also biochemical characteristics to accurately classify this organism.

## INTRODUCTION

Isolated from varied, and often extreme environments, the *Pseudomonas* genus now contains 300 different species that have been validated, excluding subspecies and synonymous species (1) (https://lpsn.dsmz.de/genus/pseudomonas, accessed on 22 September 2022). The *Pseudomonas* genera is divided into 16 phylogenetic groups (2), of which currently *Pseudomonas fluorescens* is the largest (3). The *P. fluorescens* group is further split into 11 subgroups comprising of *P. asplenii*, *P. chlororaphis*, *P. corrugata*, *P. fluorescens*, *P. fragi*, *P. gessardii*, *P. jessenii*, *P. koreensis*, *P. mandelii*, *P. protegens*, and *P. kielensis* (2). The *P. fluorescens* complex continues to grow with a number of novel species being added to this group over the last decade (2, 4, 5).

Strains within the *P. fluorescens* complex are metabolically diverse, allowing them to survive in a wide variety of environments (6). While most of the species within the *P. fluorescens* complex are plant-associated bacteria, more recently, some strains have been known to cause opportunistic infections in humans and have been isolated from hospital environments (6 – 8). Besides being able to survive on different nutrient resources, these strains are also resistant to several industrial and medically important antibiotics (9 – 12). *P. fluorescens* complex strains differ markedly in their biochemical characteristics and often relying solely on 16s rRNA profiling for classification does not provide the granularity required to accurately differentiate species within this closely related complex (13, 14). The most recent example is the classification of *Pseudomonas allokribbensis* sp. nov. and *Pseudomonas gozinkensis* sp. nov. from a volcanic island in Japan (4). The two strains, belonging to the *P. fluorescens* complex, had >99% 16s rRNA sequence identity with the closest type strain, *Pseudomonas kribbensis*. However, phylogenetic analysis based on whole-genome sequencing, average nucleotide identity (ANI) scores (93.1% and 90.9%), and DNA-DNA hybridization (DDH) scores (51% and 43.2%) compared to the type strain demonstrated that the strains were, in fact, two novel species. In addition to genotypic characterization, a thorough phenotypic analysis helps distinguish strains, especially when they are genetically very similar to each other. Phenotypic analysis, such as biochemical characterization, can help identify strains that could be used in industrial or environmental applications.

In this study, we isolated a strain from a sealed vial of lyophilized hygromycin B, an antibiotic routinely used in research laboratories. Hygromycin B is an aminoglycoside antibiotic not used clinically due to its cytotoxicity toward mammalian cells but is often used in research laboratories because of its activity against fungi, higher eukaryotic cells, and bacteria, including many highly antibiotic-resistant bacteria (15). The discovery of the resistant strain occurred while using hygromycin B for selection in cloning experiments. We performed whole-genome sequencing of the strain and characterized it using various methods with the aim to understand its genetic and phenotypic profile. We potentially identified the gene responsible for its resistance to the antibiotic and show that the strain is not a human pathogen but can survive at low temperatures, infect plants and insects, and compete with *Escherichia coli*. Based on the results of this study, we propose a novel species, *Pseudomonas hygromyciniae* sp. nov. (type strain SDM007^T^).

## RESULTS

### Isolation of a resistant strain from a vial of lyophilized hygromycin B

A sealed bottle of lyophilized hygromycin B was opened, and a portion of the powder was aliquoted into sterile lysogeny broth (LB), which was then stored at 4°C. When the LB containing hygromycin B was retrieved from the refrigerator after several days, it contained visible microbial growth. Distinct and uniform colonies were observed when the medium was streaked onto LB agar plates with or without 500 µg/mL filter-sterilized hygromycin B. A single colony was picked from a hygromycin plate and regrown in sterile LB medium containing 500 µg/mL filter-sterilized hygromycin B, and a frozen stock was made for use in all subsequent studies. The colonies that grew on the LB agar plates were off-white and of a single morphology (Fig. S1A) and had a pungent odor. These colonies were fluorescent under a 302 nm blacklight (Fig. S1B). The organism grew into visible colonies on plates incubated for 24–48 h at 4, 22, and 30°C but not at 37°C; growth rate based on colony size was highest between 22 and 30°C. Microscopy indicated that the organism was a gram-negative rod-shaped bacterium of approximately 1.5 µm in length and 0.5 µm in width (Fig. S1C).

### SDM007^T^ belongs to the *Pseudomonas fluorescens* complex

Genomic characterization of SDM007^T^ was undertaken with Illumina (short-read) and Nanopore (long-read) sequencing. Sequencing reads were assembled *de novo* to yield a complete, closed genome. The resulting sequence consisted of 6,037,020 bp circular chromosome with a G + C content of 60.2% and a 249,868 bp circular megaplasmid with a G + C content of 55.3%. A BLAST analysis of the SDM007^T^ genome against the NCBI 16s rRNA gene sequence database for bacteria and archaea indicated that it has a 16s rRNA sequence most closely related to those of *Pseudomonas gessardii* (99.67% identity) and *Pseudomonas libanensis* (99.67% identity) (Supplementary Information 1) indicating that the strain falls within the *P. fluorescens* complex. *In silico* DDH (16) was performed using the Type Strain Genome Server (TYGS) (17) to compare SDM007^T^ to the type strains in TYGS. A *d*_4_ value of ≤70% to the next nearest genome has been considered one potential indicator of a distinct species (18). SDM007^T^ did not belong to any species found in the TYGS database, and the strain with the highest predicted DDH to SDM007^T^ was *Pseudomonas proteolytica* strain LMG 22710 ^T^ (*d*_4_ of 60.1%).

**Fig 1:**
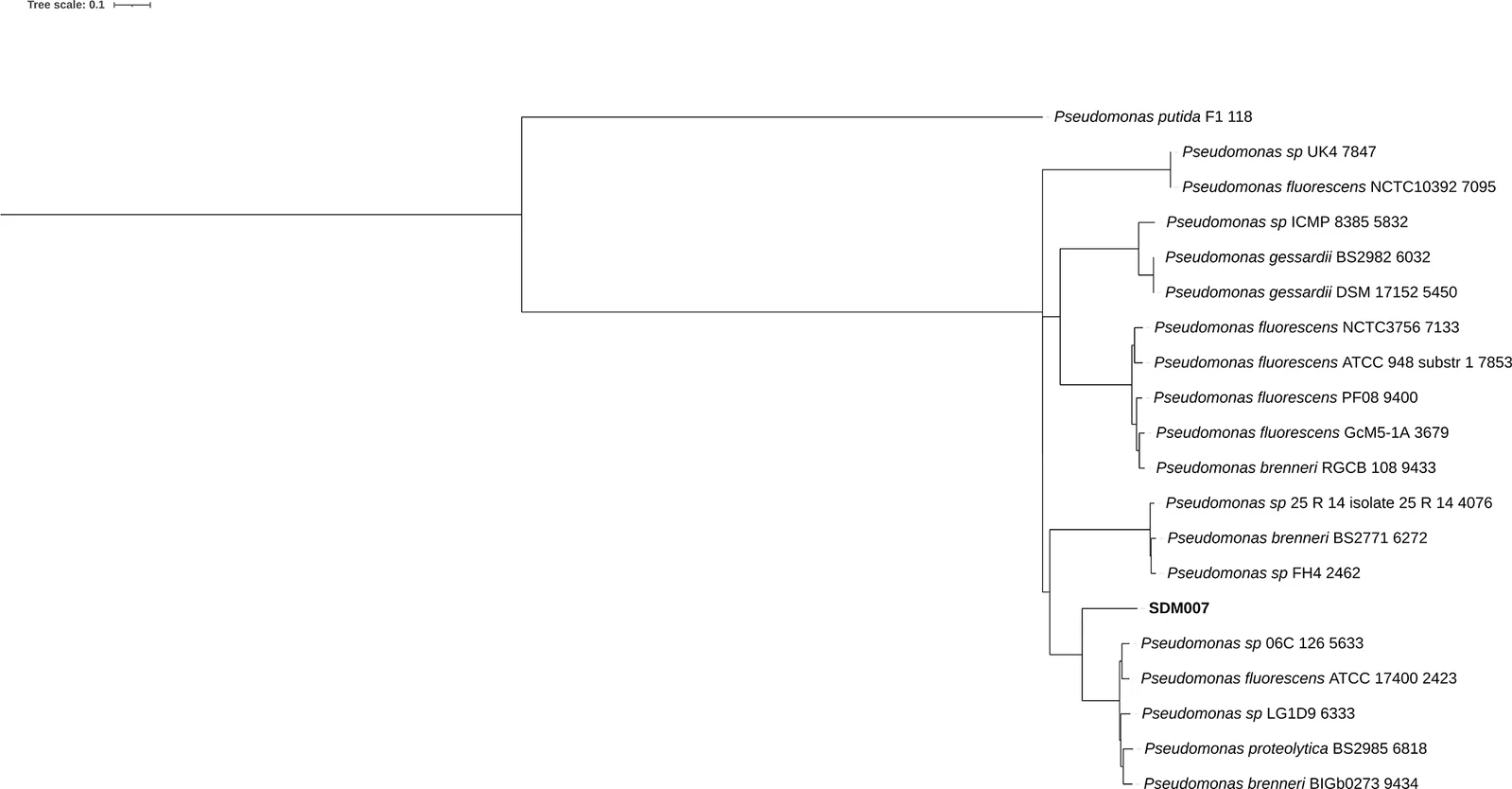
Phylogeny of SDM007^T^ and closely related bacteria. A phylogenetic tree was generated based on SNP loci occurring in at least 95% of the analyzed genomes. *Pseudomonas putida* reference strain F1 was included to root the tree.

Besides *in silico* DDH, an ANI difference of ≤95%–96% has also been used to distinguish species (18). An approximate ANI of SDM007^T^ with 9,108 publicly available *Pseudomonas* spp. genomes was calculated using a previously published nucleotide similarity script (19). The 10 most similar genomes from this analysis were then compared to SDM007^T^ (Table 1) and to each other (Table S1) using the JSpeciesWS ANI-calculating software (20). Comparing SDM007^T^ to *P. brenneri* BIGb0273 gave an ANIb value of 94.41% with 86.78% aligned nucleotides, which while highly similar still falls below the ANI cut-off value with a high percentage of aligned nucleotides. Comparing genomes of *P. brenneri* BIGb0273, *P. fluorescens* ATCC17400, and *P. proteolytica* BS2985 to each other gave high ANIb values of 97.65%–98.35% (Table S1). Next, a parsimony-based phylogenetic tree was generated from SNP loci occurring in at least 95% of the genomes of SDM007^T^ and 18 closely related *Pseudomonas* strains based on ANI. *P. putida* reference strain F1 was included as an outgroup to root the tree. The midpoint-ooted tree illustrated the close relationship between SDM007^T^ and *P. brenneri*, *P. proteolytica*, and *P. fluorescens* and placed the type strain in a separate lineage (Fig. 1).

**TABLE 1:** Average nucleotide identity and the aligned nucleotide percentage of the 10 most closely related species compared to *P. hygromyciniae* sp. nov. *strain SDM007^T^*

	ANIb (%) compared to SDM007^T^	Aligned nucleotide percentage
*P. brenneri* BIGb0273	94.41	86.78
*Pseudomonas* sp. LG1D9	94.34	82.39
*P. fluorescens* ATCC17400	94.00	82.14
*P. proteolytica* BS2985	93.92	80.51
*P. brenneri* BS2771	90.66	78.27
*Pseudomonas* sp. FH4	90.52	78.08
*Pseudomonas* sp. 25R14	90.80	76.09
*P. fluorescens* NCTC10392	90.59	72.37
*P. gessardii* DSM17152	90.54	72.11
*Pseudomonas* sp. ICMP8385	90.37	70.59

Together, the 16s rRNA gene sequencing, DDH, ANI, and phylogenetic tree results support the strain being a novel species, which we propose to name *Pseudomonas hygromyciniae* sp. nov. with SDM007^T^ as the type strain, and have further characterized.

### Phenotypic characteristics of SDM007^T^

**Fig 2:**
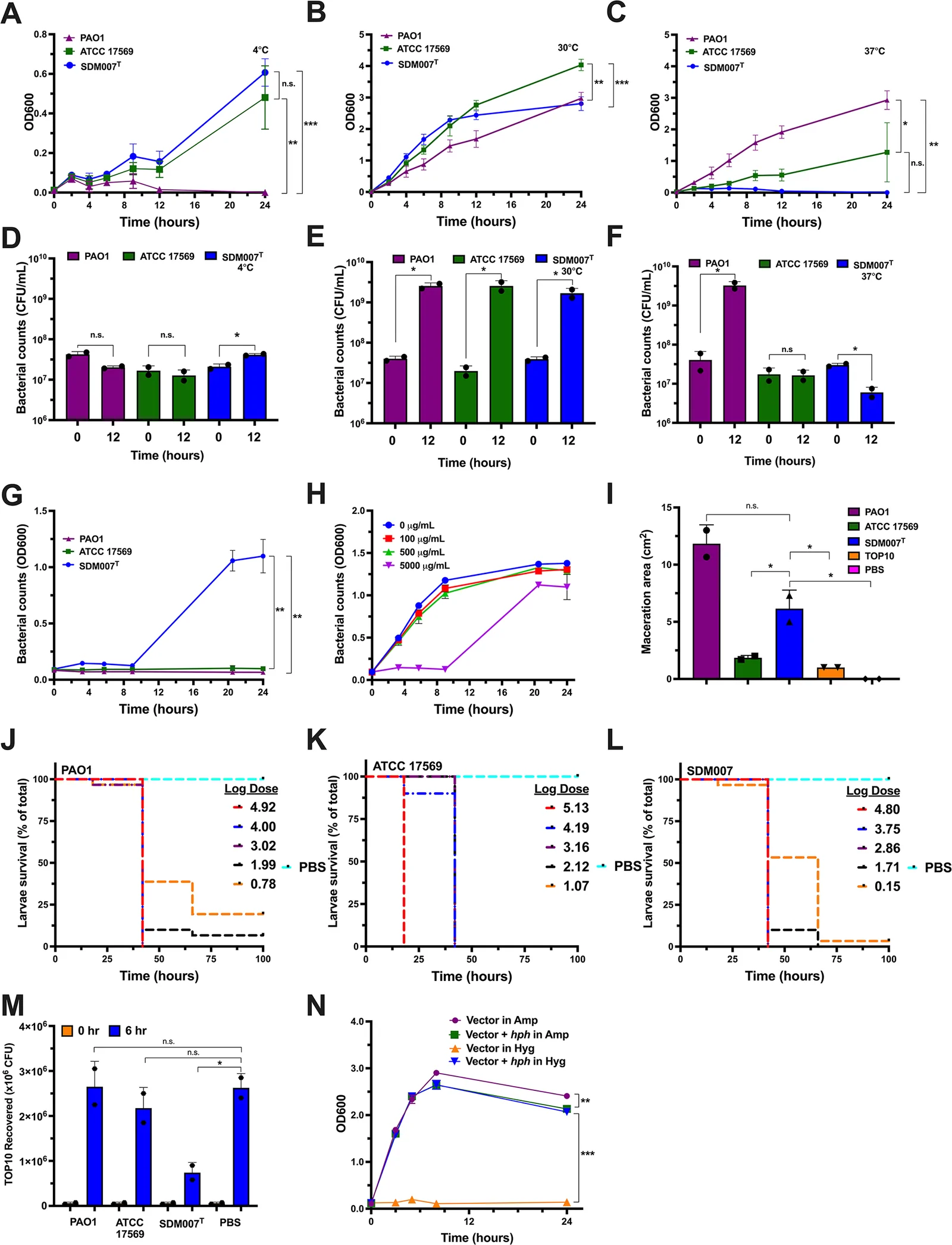
Phenotypic characterization of *P. hygromyciniae* sp. nov. *strain SDM007^T^*. Optical density (OD_600_) values of SDM007, *P. fluorescens* strain ATCC 17569, and *P. aeruginosa* strain PAO1 in LB medium at (**A**) 4°C, (**B**) 30°C, and (**C**) 37°C over 24 h. CFU in LB medium at (**D**) 4°C, (**E**) 30°C, and (**F**) 37°C over 12 h. (**G)** OD_600_ values of strains grown in LB medium supplemented with 500 µg/mL hygromycin B at 30°C. (**H)** OD_600_ values of SDM007^T^ grown for 24 h in LB medium containing 0; 100; 500; or 5,000 µg/mL hygromycin B at 30°C. At 24 h, the differences in CFU were not significant. (**I)** Area of maceration of romaine lettuce leaves inoculated with SDM007^T^, ATCC 17569, PAO1, *E. coli* strain TOP10, and PBS. Survival of *G. mellonella* larvae when infected with five doses of (**J**) PAO1, (**K**) ATCC 17569, or (**L**) SDM007^T^. (**M)** TOP10 CFU recovered after 6 h co-incubation with PAO1, ATCC 17569, SDM007^T^, or PBS. (**N)** Growth over 24 h of *E. coli* TOP10 expressing the *hph* gene cloned from SDM007^T^. “Vector” refers to the pEX18.AP plasmid (which contains an ampicillin-resistance cassette) with or without the *hph* genes. “in Amp” or “in Hyg” refers to growth in ampicillin or hygromycin, respectively. Ordinary one-way ANOVA was used to compare the means across the end timepoints in a–c, g, h, and n; otherwise, *t*-test was used to determine statistical difference. *P < 0.05, **P < 0.01, and ***P < 0.001.

To better understand how SDM007^T^ may differ from other *Pseudomonas* spp., a variety of phenotypic assays were conducted to compare this bacterium to *Pseudomonas aeruginosa* and *P. fluorescens*. SDM007^T^ grew at a rate comparable to the *P. fluorescens* strain ATCC 17569 at 4°C, while *P. aeruginosa* strain PAO1 did not show appreciable growth at this temperature (Fig. 2A). At 30°C, SDM007^T^ and *P. aeruginosa* PAO1 showed similar growth kinetics, and *P. fluorescens* ATCC 17569 grew slightly faster (Fig. 2B). Notably, SDM007^T^ failed to grow at 37°C after 24 h unlike *P. fluorescens* ATCC 17569 and *P. aeruginosa* PAO1 (Fig. 2C). Although SDM007^T^ survives at 4°C (Fig. 2D) and has comparable growth to the control strains at 30°C (Fig. 2E), it rapidly died at 37°C, with a decrease of approximately 1 log of viable CFUs after 12 h of incubation (Fig. 2F). Unsurprisingly, SDM007^T^ is resistant to hygromycin B, growing from an initial OD_600_ of ~0.1 to over 1 within 20 h when incubated at 30°C in LB containing 500 µg/mL of hygromycin (Fig. 2G). Subsequent tests indicated that it is capable of growing in LB with much higher concentrations of hygromycin. No statistically significant difference in OD_600_ was observed at the 24 h timepoint when grown in LB medium supplemented with 500 µg/mL vs 5 mg/mL hygromycin B although the growth rate was slower over the first 8 hr (Fig. 2H).

Because this strain was isolated from a vial of hygromycin B, we investigated whether this bacterium used hygromycin B as a carbon or nitrogen source. SDM007^T^ was grown in M9 minimal medium with or without carbon or nitrogen sources supplemented with hygromycin B. Although it grew in complete M9 medium supplemented with hygromycin, it was unable to grow in the same medium lacking glucose (carbon) or ammonium chloride (nitrogen), indicating that it cannot use hygromycin B as a carbon or nitrogen source (Fig. S2). We next examined carbon sources that could be used by SDM007^T^ for growth (Table S2). Of 190 tested carbon sources, it showed measurable growth on 74 (39%), including sugars (glucose, fructose, galactose), amino acids (L-alanine, L-arginine, L-histidine), fatty acids (capric acid, propionic acid), and nucleosides (adenosine, uridine). On comparing it with its closest relative, *P. brenneri* (type strain DSM15294) (21, 22), we found that SDM007^T^ was unable to metabolize L-rhamnose and adonitol. Conversely, it was able to metabolize L-histidine, L-ornithine, and fumaric acid while the type strain *P. brenneri* DSM15294 could not. These results indicate that SDM007^T^ is metabolically versatile and may be able to grow in a variety of environments, with its primary limitation being the requirement for temperatures below 37°C.

### SDM007^T^ is a potential plant pathogen

Since several bacteria within the *P. fluorescens* group are associated with the rhizosphere, we investigated whether SDM007^T^ was pathogenic toward plants using a lettuce leaf model (23). We inoculated the strain into the midribs of romaine lettuce leaves. After 72 h, it displayed an area of maceration (AoM) of 6.17 cm^2^, which was smaller than the 11.83 cm^2^ AoM of the known plant pathogen *P. aeruginosa* PAO1 (24) but larger than the AoMs of *P. fluorescens* ATCC 17569, *Escherichia coli* TOP10, and PBS (1.83 cm^2^, 1.0 cm^2^, and 0.0 cm^2^, respectively; Fig. 2I). These results suggest that SDM007^T^ is a potential plant pathogen.

### SDM007^T^ is virulent to *Galleria mellonella* but not in A549 cells

We next examined the virulence of SDM007^T^ in a *Galleria mellonella* (greater wax moth), an infection model used to assess virulence of *P. aeruginosa* strains (25). When injected into *Galleria* larvae, killing by *P. aeruginosa* PAO1 (Fig. 2J) and *P. fluorescens* ATCC 17569 (Fig. 2K) were similar to that of SDM007^T^ at room temperature. Specifically, it killed nearly all larvae within 72 h. Even at the lowest inoculum of 1.4 CFU (log 0.15), only a single larva out of 30 survived (Fig. 2L). These results indicate that SDM007^T^ *,* similar to the other *Pseudomonas* species tested, is highly virulent in the *Galleria* model.

*P. aeruginosa* is a prominent human pathogen, and *P. fluorescens* has caused outbreaks among hospitalized patients (8). For this reason, we examined the virulence of SDM007^T^ in a mammalian system despite its inability to grow at 37°C. We incubated the strain with A549 lung carcinoma epithelial-like cells and measured cytotoxicity. *P. aeruginosa* PAO1 caused 10% lysis of the cells after 8 h at 37°C, but SDM007^T^ and *P. fluorescens* ATCC 17569 displayed only ~1% and 0% cell lysis, respectively (Fig. S3). Given that SDM007^T^ rapidly dies at 37°C, supernatants from overnight cultures of SDM007^T^ grown at 30°C were spiked into A549 wells and allowed to incubate for 8 h, but no cell killing was detected above the level of background (Fig. S3). These data suggest that the strain is unlikely to be a human pathogen as it does not proliferate at normal physiological temperatures, does not kill a representative mammalian cell line, and does not appear to secrete virulence factors capable of eliciting death of this cell line.

### SDM007^T^ competes with *E. coli*

Since a rich variety of bacteria inhabit the soil, these bacteria frequently harbor mechanisms to compete against each other. To explore whether SDM007^T^ can successfully compete with other bacteria, a competition assay with *E. coli* was conducted. SDM007^T^ and *E. coli* TOP10 were mixed in liquid culture, plated at high density on LB agar plates, and incubated for 6 h at 30°C. *E. coli* CFU were then enumerated by plating on selective agar. Fewer than half the number of *E. coli* CFU were recovered from the SDM007^T^ plates compared to the *P. aeruginosa* or *P. fluorescens* plates (Fig. 2M). These findings indicate that SDM007^T^ has evolved mechanisms that allow it to successfully compete against some other bacteria.

### pSDM007 confers hygromycin resistance through hph gene

We next focused on the 250 kb plasmid, designated “pSDM007,” found in SDM007^T^. Read coverage analysis indicated ~1.4 copies of pSDM007 per genome, suggesting that this is a single-copy plasmid in majority of cells. A search of the pSDM007 sequence against all Gammaproteobacteria in the NCBI database (taxid: 1236) as of December 29, 2020, yielded a top hit of the *Pseudomonas rhodesiae* BS2777 genome (44% coverage and 98.20% identity) and the *P. aeruginosa* plasmid pNK546b (36% query cover and 80.57% identity) (Supplementary Information 2). The plasmid contains several genes predicted to play a role in conjugation and replication of plasmids (Fig. S4; Table S3).

pSDM007 contained two genes predicted to confer resistance to aminoglycosides: *hph,* a known hygromycin-B 4-O-kinase; and an uncharacterized gene [72.57% identity to AAC(2′)-IIa, an aminoglycoside resistance gene] by the Comprehensive Antibiotic Resistance Database (26). To examine whether either were responsible for the hygromycin B resistance exhibited by SDM007^T^, each of the two genes along with their promoter regions was cloned into isopropylthiogalactoside (IPTG)-inducible expression plasmids. These plasmids were transformed into *E. coli* TOP10 cells, which were then grown in the presence or absence of hygromycin. Resistance to hygromycin B was not conferred by the AAC(2′)-IIa-like gene in either the presence or absence of IPTG (Fig. S5). However, the *hph* gene did confer growth in the presence of hygromycin B (100 µg/L) even without IPTG induction (Fig. 2N). These results indicate that the *hph* gene carried by the pSDM007 plasmid was at least partially responsible for the hygromycin resistance of SDM007^T^.

### Reisolating *P. hygromyciniae* sp. nov. from another vial of hygromycin B

We examined whether *P. hygromyciniae* sp. nov. could be cultured from other commercially available preparations of hygromycin B. A second bottle of hygromycin B was purchased from the same vendor as the original vial. This second vial was from the same lot as the original isolation source. Hygromycin B was also purchased from two other vendors. When unfiltered hygromycin B from each stock was added to individual culture tubes of liquid LB and cultured at room temperature for 72 h, bacterial growth only occurred from the preparation purchased from the original vendor. DNA from a single colony of this growth (designated SDM007_2) was sequenced, and the resulting genome was found to be similar to the original SDM007^T^ genome, differing by ~700 SNVs on the chromosome and by zero SNVs on the pSDM007 plasmid. These findings confirm that the hygromycin vials were, indeed, the source of *P. hygromyciniae* sp. nov. and that it was not acquired by contamination in our laboratory. They also suggest that a non-clonal population of this bacterium exists at some point in the hygromycin B production chain of this vendor.

## DISCUSSION

The *Pseudomonas* genus consists of ubiquitous microbes found in wide-ranging environments across the globe. Their genetic diversity is vast, even within single species, such as *P. fluorescens*. This genetic diversity can be at least partially owed to the ability for *P. fluorescens* to adapt to extreme environments (6), and here, we have shown that this adaptation has allowed for survival in a commercial, lyophilized antibiotic. To our knowledge, this is the first instance of a novel microbial species being identified from commercial antibiotics. This discovery opens the door for more thorough investigations for potential microbial discoveries from different classes of manufactured antibiotics.

With the current phenotypic and genotypic characterization tools, especially whole-genome sequencing, the pipeline for taxonomic classification calls for a more holistic approach. With this in mind, we genetically characterized SDM007^T^ using 16s rRNA profiling, *in silico* DDH against type strains in TYGS, and ANI. The strain showed high 16s rRNA sequence identity with *P. gessardii* and *P. libanensis* (99.67%). The 16s rRNA sequence between closely related *Pseudomonas* species can differ by <1%, and high 16s rRNA sequence identity has previously been reported not only for species within the *P. fluorescens* complex (4) but also within *P. putida* complex (27) and *Brucella* (28). The strain was identified as a proposed novel species within the *P. fluorescens* complex group, being most closely related to *P. brenneri* BIGb0273.

Initially characterized as a gram-negative rod-shaped strain emitting a pungent odor and was able to grow in high concentrations of hygromycin B, up until the tested concentration of 5 mg/mL. However, the strain was unable to grow at 37°C, with or without hygromycin B. This ability to survive at 4°C, and thrive at 30°C, could be attributed to potential cold temperature adaptative mechanisms such as cold-adapted chaperonins (29), and/or the ability to use metabolites at a lower temperatures (30), or a change in the membrane composition. Given the range of temperature the strain could survive in, we characterized its ability to infect different hosts such as plants (lettuce leaf model), insects (*Galleria mellonella* model), and humans (cytotoxicity assay on A549). The proposed novel species is unlikely a human pathogen due to its inability to survive at 37°C as well as its lack of cytotoxicity toward a mammalian cell line but was able to infect plant and insect hosts, as well as outcompete *E. coli* in an *in vitro* environment.

SDM007^T^ showed metabolic diversity, utilizing 39% of the carbon sources tested but was unable to use hygromycin B as a carbon or nitrogen source, indicating an alternate resistance mechanism to the antibiotic. The 250 kb megaplasmid found in SDM007^T^ contained two genes—*hph* and an uncharacterized AAC(2′)-IIa-like gene which could potentially confer resistance to hygromycin B. Expressing these genes individually into *E. coli* Top10 showed that *hph*, which encodes a hygromycin-B 4-*O*-kinase, and not the AAC(2′)-IIa-like gene enabled the strain to grow in the presence of hygromycin B.

We propose this organism as a novel species with the name *Pseudomonas hygromyciniae* sp. nov. based on the source from which this bacterium was discovered and *Pseudomonas hygromyciniae* sp. nov. *strain SDM007^T^* as the type strain.

## MATERIALS AND METHODS

### Bacterial strains, media, and reagents

*P. hygromyciniae* sp. nov. *SDM007* ^T^ was isolated from a sealed bottle of lyophilized hygromycin B purchased from Sigma-Aldrich Co. (product number H3274-1G, lot number SLBZ3956, St. Louis, Missouri, USA) in November 2019. A second bottle of hygromycin B of the same product and lot number was purchased Sigma-Aldrich Co. in February 2020. Other hygromycin B vials were purchased from Dot Scientific, Inc. (product number DSH75020-1, lot number 94491-105646) and Gold Biotechnology (product number H-270-1). Ampicillin was purchased from Acros Organics. *E. coli, P*. aeruginosa, *P. fluorescens,* and SDM007^T^ were grown aerobically (250 RPM) in LB broth (10 g/L tryptone, 5 g/L yeast extract, 10 g/L sodium chloride) or on LB agar plates (LB broth supplemented with 16 g/L agar). M9 medium was made as follows: Na_2_HP0_4_ 6.9 g/L, KH_2_PO_4_ 3 g/L, NaCl 0.5 g/L, NH_4_Cl 1 g/L, CaCl_2_ 0.1 mM, MgSO_4_ 2 mM, and glucose 0.5%. A549 (male, human lung epithelial-like) cells were obtained from ATCC and grown at 37°C in DMEM (Gibco) supplemented with 10% FBS (GE Healthcare Life Science) under 5% CO_2_. All strains used in this study are listed in Table S4.

### Whole-genome sequencing and sequence analysis

Genomic DNA was extracted using a Maxwell 16 Cell DNA Purification Kit (Promega). Genomic DNA libraries were prepared using a Nextera XT kit (Illumina) following the manufacturer’s protocol. Short-read sequencing was performed on a MiSeq platform (Illumina) generating 300 bp paired-end reads. For SDM007^T^ 3,348,302 reads were generated totaling 826,593,557 bp with an average read length of 247 bp after adapter trimming. The average fragment size for SDM007^T^ was 804 bp, which includes adapters trimmed from the data while sequencing. Libraries were prepared for long-read sequencing using the ligation sequencing kit (SQK-LSK109, Oxford Nanopore) and were sequenced on the MinION platform JAFFTG000000000 using a FLO-MIN106D flow cell (Oxford Nanopore). Nanopore basecalling with Guppy v3.3.3 yielded 27,630 reads that passed quality filtering and totaling 216,452,583 bases with an average read length of 7,834 bp and an L50 value of 5,006. Hybrid *de novo* assembly of SDM007^T^ was performed by first assembling Nanopore long reads using Flye (v2.5) (31), which generated two circularized contigs. Next, Illumina short reads were aligned to the contigs using BWA (v0.7.17) (32) and mismatch and indel errors corrected using Pilon (v1.23) (33). Alignment and error correction were repeated until no new corrections were made by Pilon, after a total of five cycles. Prokka v1.12 (34) was used for initial annotations of the SDM007^T^ genome with additional annotations via BLAST (35) and the Comprehensive Antibiotic Resistance Database (26) analysis. A phylogenetic tree was constructed using kSNP v3.0.21 (36, 37) based on SNP loci occurring in at least 95% of genomes. Short-read sequencing of SDM007_2 was performed similarly to that of SDM007. The genomes of SDM007, SDM007_2, and plasmid pSDM007 have been deposited in the GenBank database as accession numbers CP070506, JAFFTG000000000, and NZ_CP070507, respectively.

Polymorphisms between the two *P. hygromyciniae* sp. nov. isolates were determined as previously described (38). Briefly, Illumina reads from the second isolate were trimmed with Trimmomatic (v0.36) (39) and aligned to the SDM007^T^ chromosome or plasmid using BWA (v0.7.15) (32). Variant sites meeting quality criteria and not in repetitive region of the reference sequence were then identified using SAMtools (v0.1.19-44428cd) (40) and a custom Perl script.

16s rRNA gene sequence analysis utilized the NCBI BLAST 16s ribosomal RNA sequence (bacteria and archaea) database (41). *In silico* DNA-DNA hybridization utilized the Type Strain Genome Server (17) DDH *d*_4_ value. All *Pseudomonas* spp. genomes (9,108 as of 30 December 2019) available from the *Pseudomonas* Genome Database (42) were downloaded, and a custom ANI script (19) was used to compare all genomes to SDM007. The ANIb values of the top 10 strains compared to SDM007^T^ were recalculated using JSpeciesWS (20). A parsimony phylogenetic tree was constructed using kSNP v3.0.21 (37) based on SNP loci occurring in at least 95% of the 18 analyzed genomes, including the *P. putida* reference strain F1 as the outgroup, and midpoint rooted and visualized using iTOL (43).

### Growth curves and antimicrobial susceptibility testing

For growth curves, SDM007^T^ bacteria and control bacteria from −80°C frozen stocks were struck onto LB agar plates with or without 500 µg/mL hygromycin B. Individual colonies were grown overnight in liquid LB medium at 30°C with shaking and sub-cultured for 3 h in LB. All strains were adjusted to a final optical density at 600 nm (OD_600_) of 0.10 and inoculated into 5 mL of culture media. Cultures were incubated at 4, 30, and 37°C in 250 RPM orbital shakers (Thermo Fisher MaxQ 4000, USA). Samples were taken at set timepoints and diluted if needed. OD_600_ values were obtained by placing 200 µL of each sample into a 96-well plate on a SpectraMax M3 plate reader (Molecular Devices) and measuring OD_600_. Bacterial CFUs were assessed by serial dilutions of samples and plating onto LB agar plates for enumeration of colonies after overnight growth at room temperature.

To determine growth rates of strains in the presence of hygromycin B, bacteria were grown as described above except that 0; 100; 500; or 5,000 µg/mL of filter-sterilized hygromycin B were added to the medium. Bacteria were added to these hygromycin-supplemented media to obtain a final OD_600_ of 0.10. A total of 200 µL of the bacterial suspensions were added to a Costar 96-well clear, flat-bottom plate (Corning). Cultures were incubated for up to 48 h at 30°C with periodic OD_600_ measurements.

Whether SDM007^T^ could utilize hygromycin as a carbon or nitrogen source was tested using standard M9, M9 without ammonium chloride, and M9 without glucose media. Bacterial suspensions in the indicated medium were adjusted to an OD_600_ of 0.10, and 200 µL was added to a Costar 96-well, clear, flat-bottom plate. Cultures were incubated for 24 h at 25°C with 250 RPM shaking and periodic reading of OD_600_ values using a SpectraMax M3 plate reader.

All growth curves, antibiotic resistance assays, and minimal media assays were performed with at least two technical replicates (per biological replicate) and three biological replicates, and results are shown as means of these data.

### Expression of *hph* and AAC(2′)-IIa-like gene in *E. coli*. TOP10

Oligonucleotides were purchased from Integrated DNA Technologies (Iowa, USA). Polymerase chain reactions (PCR) were performed using Phusion High-Fidelity DNA Polymerase (New England Biolabs), and gel-purified products [*hph* and the gene of unknown function with 72.57% homology to an AAC(2′)-IIa resistance gene] were cloned into the pEX18.AP vector using the Gibson Assembly Cloning Kit (New England Biolabs) per the manufacturer’s instructions. Chemical competent *E. coli* TOP10 were transformed with the Gibson construct following standard protocol and were plated on LB agar plates containing 100 µg/mL ampicillin. Overnight colonies were selected, and the transformed construct underwent PCR; the gel-purified product was Sanger sequenced through the Northwestern University NUSeq Sanger Sequencing Core for sequence confirmation. TOP10 harboring a pEX18.AP empty vector control was also transformed using the above protocol without addition of the gel-purified products.

Freezer stocks of the strains were grown overnight at 37°C onto LB agar with 100 µg/mL ampicillin. A single colony of each strain was picked into 5 mL LB media with 100 µg/mL ampicillin and grown overnight at 37°C in a 250 RPM orbital shaker. A 500 µL aliquot of the overnight culture was transferred to 5 mL of fresh LB with 100 µg/mL ampicillin and grown until mid-log phase. The resulting culture was centrifuged briefly, and the cell pellet was washed in sterile PBS twice and then resuspended in 1 mL of PBS. The resuspended cells were aliquoted into 5 mL of LB with 100 µg/mL ampicillin or LB with 100 µg/mL hygromycin B for a final OD_600_ of 0.1. Optical densities were monitored for overtime to determine if the cloned *hph* and AAC(2′)-IIa-like genes provided hygromycin B resistance in TOP10.

All plasmids and primers used in this study are listed in Table S5 and S6, respectively.

### Lettuce model of infection

Lettuce leaf infection assays were conducted following the protocol of Starkey and Rahme (23) with minor modifications using store-bought Earthbound Farm’s Organic Whole Romaine Leaves (lot #TFRB024B4A, product of Mexico). A 5-µL-aliquot of bacteria adjusted to an OD_600_ of 1 was injected into the midrib of the lettuce, and inoculum size was verified by plating and CFU enumeration. Petri dishes containing the inoculated leaves and Whatman no. 1 filter paper soaked in 10 mM MgSO_4_ were individually placed along with a paper towel soaked in 10 mM MgSO_4_ into partially sealed 3.78 L storage bags (Target Corporation). Storage bags containing the petri dishes were then incubated at 30°C. After 72 h, areas of necrosis were measured. All tests were performed with at least three technical replicates (per biological replicate) and three biological replicates, and results are shown as means of these data.

### Carbon utilization

Growth with different carbon sources was assessed using Phenotypic MicroArray PM1 and PM2 MicroPlates (Biolog) following the manufacturer’s protocol. Optical densities were read using a SpectraMax M3 plate reader after 12 h of growth. Results are shown as the average of two biological and two technical replicates. OD_410_ values below 0.200 were considered to be non-utilizable carbon sources.

#### *Galleria mellonella* model of infection

Overnight bacterial cultures were inoculated into fresh LB medium and grown for 1 h and 45 min at 30°C with shaking. Cultures were pelleted, washed with sterile PBS, and diluted to provide final inoculums of approximately 10; 100; 1,000; 10,000; and 100,000 bacteria in 10 µL inoculums. Bacterial inoculums were verified by plating and CFU enumeration. The 10 µL inoculums were injected into the distal proleg of *G. mellonella* larvae (purchased from Vanderhorst Wholesale, Inc.) weighing between 200 and 350 mg using a LEGATO 100 Syringe Pump (KD Scientific) equipped with an EXEL 29.5-gauge 0.5 mL syringe (Fisher Scientific). Larvae showing immediate discharge of hemolymph or exaggerated movements post-injection were discarded. Larvae were placed in petri dishes and monitored at room temperature for up to 100 h. Larvae that did not move in response to external stimuli were scored as dead. Ten *G. mellonella* larvae were infected per condition, and experiments were repeated three times. Cumulative results of all three experiments are shown.

### Bacterial competition assay

Predator strains (SDM007^T^, *P. aeruginosa* PAO1, and *P. fluorescens* ATCC 17569) and a prey strain (*E. coli* TOP10) were grown overnight on LB agar plates at 30°C. A single colony was picked for each strain and inoculated into individual tubes of LB (5 mL), which were grown overnight at 30°C with shaking (250 RPM). Aliquots of 500 µL were then subcultured into 5 mL of LB and grown at 30°C with shaking (250 RPM) for 3 h. Cultures were spun down, washed twice with sterile PBS, and adjusted to OD_600_ ~1. A predator and a prey strain were combined at a 5:1 ratio and briefly vortexed. A total of 25 µL of the bacterial mixture was spotted onto LB agar plates, which were incubated for 6 h at 30°C. The 0 and 6 h spottings were scraped from the plate, separately resuspended in 100 µL of sterile PBS, and serially diluted onto LB plates containing LB with or without 5 µg/mL irgasan (for counter-selection against *E. coli*). At least two biological replicates each with three technical replications were performed for each experiment.

### Cytotoxicity assay

Cytotoxicity was quantified by measuring lactate dehydrogenase (LDH) release. A549 cells were seeded into 96-well polystyrene tissue culture plates (Corning) in 200 µL DMEM with phenol red and 10% fetal bovine serum. A549 cells were allowed to grow over 22 h, providing approximately 40,000 A549 cells per well. Cells were washed with PBS, and sterile RPMI medium 1640 (Gibco) was added to each well. Bacterial strains were cultured overnight in LB medium with or without hygromycin B (500 µg/mL). Aliquots of the overnight culture were pelleted by centrifugation at 21,130 × *g* for 90 s and washed with PBS twice. Bacteria were resuspended in PBS and added to each A459-containing well at a final multiplicity of infection (MOI) of 1. PBS alone was added to some wells to measure background LDH release, and 1% Triton X-100 in PBS to other wells to measure total cell lysis. The 96-well plate was centrifuged at 500 × *g* for 5 min to enhance bacteria-to-cell contact. Infected cells were incubated at 37°C under 5% CO_2_. After 8 h, the plate was spun at 500 × *g* for 5 min to pellet cell debris, and 30 µL of supernatant was removed from the top of each well for measurement of LDH release using a Pierce LDH Cytotoxicity Assay Kit (Thermo Scientific) as per manufacturer’s instructions. Cytotoxicity was calculated as a percentage of total cell lysis (wells with Triton X-100 in PBS) as follows: % cytotoxicity = [(Sample OD_490_ – Sample OD_680_) – (PBS OD_490_ – PBS OD_680_)]/[(Triton X-100 OD_490_ – Triton X-100 OD_680_) – (PBS OD_490_ – PBS OD_680_)] × 100. Tests were performed with at least two biological replicates, each with three technical replicates, and results are shown as means of these data.

### Statistical analysis

All experiments were conducted with at least two biological replicates consisting of at least two technical replicates. Error bars indicate standard errors for a given experiment. *P*-values were calculated by either ordinary one-way ANOVA with Tukey multiple comparison test or two-tailed student *t*-tests with the help of GraphPad by Prism or the Analysis ToolPak addon (Microsoft Excel).

## Data Availability

The genomes of SDM007, SDM007_2, and plasmid pSDM007 have been deposited in the GenBank database as accession numbers CP070506, JAFFTG000000000, and NZ_CP070507, respectively. *Pseudomonas hygromyciniae* sp. nov. strain SDM007^T^ has been deposited at the American Type Culture Collection (ATCC) as deposit TSD-353 and at the Belgian Coordinated Collections of Microorganisms (BCCM) as deposit LMG 32766.

## SUPPLEMENTAL MATERIAL

Data Set S1 spectrum.01838-21-s0001.csv 16s BLAST.

Data Set S2 spectrum.01838-21-s0002.csv pSDM007 NCBI search.

Fig. S1 to S5 and Tables S1 to S6 spectrum.01838-21-s0003.docx All supplemental figures and tables.

ASM does not own the copyrights to Supplemental Material that may be linked to, or accessed through, an article. The authors have granted ASM a non-exclusive, world-wide license to publish the Supplemental Material files. Please contact the corresponding author directly for reuse.
