# Supplementary material for: Taxonomic characterization of Pseudomonas hygromyciniae sp. nov., a novel species discovered from a commercially purchased antibiotic
Source: Microbiol Spectr. 2023 Sep 22;11(5):e01838-21. doi: 10.1128/spectrum.01838-21 (PMC10581066; doi:10.1128/spectrum.01838-21)
Supplement: Fig. S1 to S5 and Tables S1 to S6 — All supplemental figures and tables. [file spectrum.01838-21-s0003.docx]

**Supplementary Information**

**Taxonomic characterization of *Pseudomonas hygromyciniae sp. nov.*, a novel species discovered from a commercially purchased antibiotic**

Timothy L. Turner^a#+^, Sumitra D. Mitra^a+^, Travis J. Kochan^a^, Nathan B. Pincus^a^, Marine Lebrun-Corbin^a^, Bettina Cheung^a^, Samuel W. Gatesy^c^, Tania Afzal^b^, Sophie Nozick^a^, Egon A. Ozer^c^, and Alan R. Hauser^a,c^

^a^Department of Microbiology-Immunology, Northwestern University, Feinberg School of Medicine, Chicago, Illinois, USA.

^b^Department of Biology, Northeastern Illinois University, Chicago, Illinois, USA.

^c^Department of Medicine, Northwestern University, Feinberg School of Medicine, Chicago, Illinois, USA.

Running title: Characterization of *P. hygromyciniae sp. nov.*

#Address correspondence to tlturner88@gmail.com

^+^ Timothy L. Turner and Sumitra D. Mitra contributed equally to this work. Author order was determined after discussion between the two first authors.

Present addresses:

Timothy L. Turner: Abbot, Lake County, Illinois, USA

Sumitra D. Mitra: Excerpta Medica B.V., Amsterdam, NL

Travis J. Kochan: United States Food & Drug Administration, Silver Spring, Maryland, USA

Nathan B. Pincus: Department of Medicine, Stanford University, California, USA


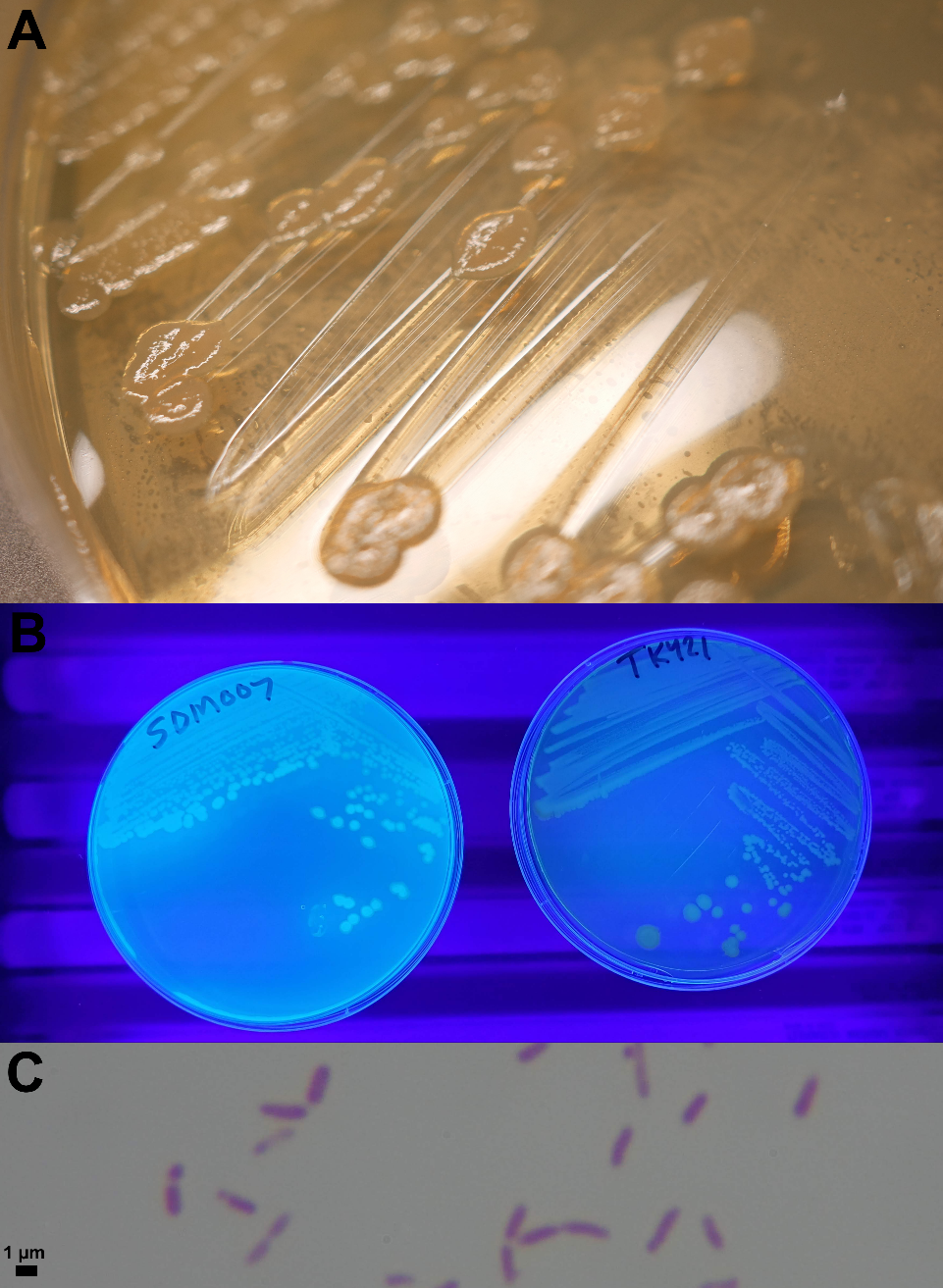


**Supplementary Figure 1.** Gram stain and colony phenotypes of *P. hygromyciniae sp. nov. strain SDM007^T^*. (A) Morphology of SDM007^T^ grown on an LB agar plate. (B) Colony fluorescence of SDM007^T^ (left) and a non-fluorescing control bacterium *Klebsiella pneumoniae* (right) under a UV light. (C) Light microscope image of SDM007^T^ after gram staining, 200x magnification.


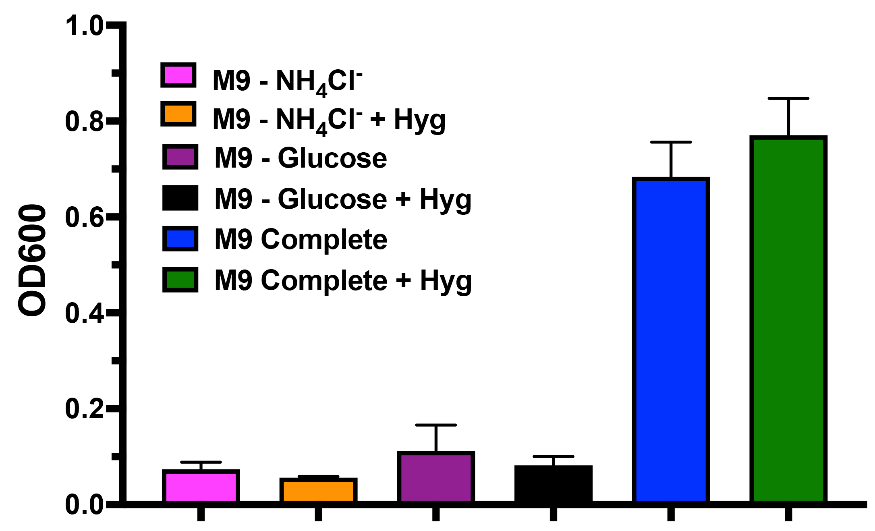


**Supplementary Figure 2.** *P. hygromyciniae sp. nov. strain SDM007^T^* growth in minimal media with or without hygromycin B. M9 minimal medium includes ammonium chloride as a sole nitrogen source and glucose as a sole carbon source. OD_600_ values are shown of SDM007^T^ after growth for 48 h in M9, M9 supplemented with hygromycin, M9 lacking ammonium chloride, M9 lacking ammonium chloride and supplemented with hygromycin, M9 lacking glucose, and M9 lacking glucose and supplemented with hygromycin. Error bars indicate standard error from two independent experiments (n=2).


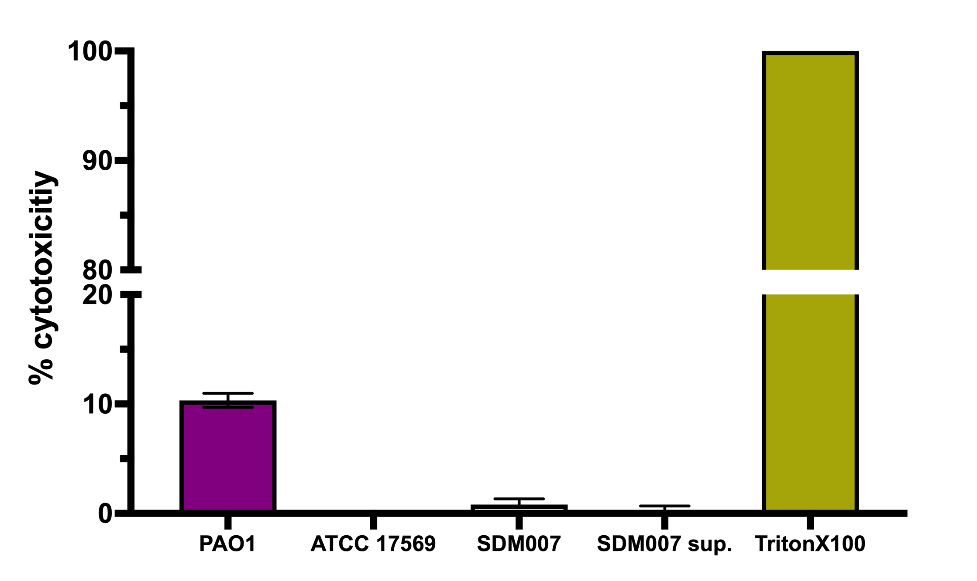


**Supplementary Figure 3.** Cytotoxicity of *P. hygromyciniae sp. nov. strain SDM007^T^* towards A549 cells. LDH release from A549 cells incubated for 8 h at 37 °C with *P. aeruginosa* strain PAO1, *P. fluorescens* strain ATCC 17569, SDM007^T^, or the supernatant of an overnight SDM007^T^ culture.


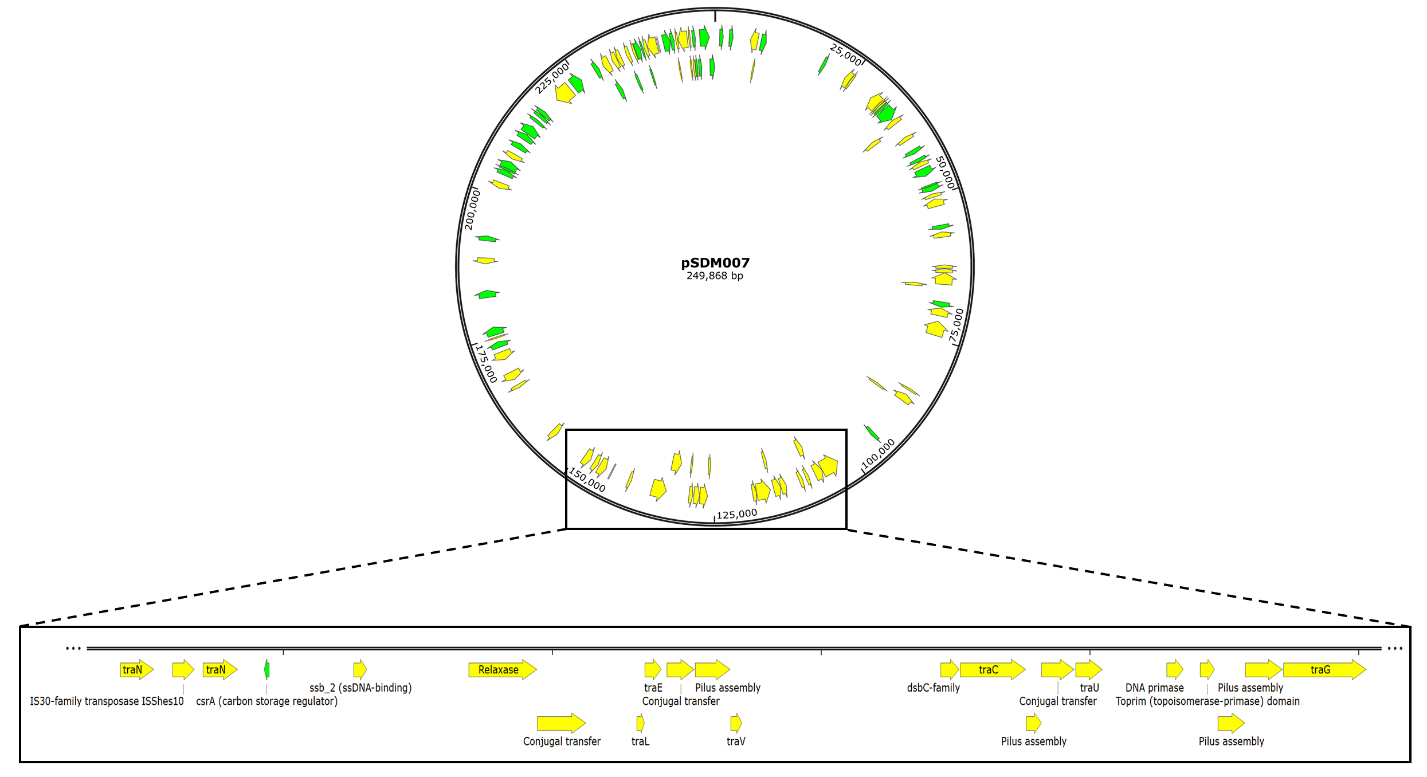


**Supplementary Figure 4.** Open reading frames of plasmid pSDM007. The pSDM007 plasmid is 250 Kbp and contains a ~45 Kbp region of putative conjugation genes (see **Supplementary Table 3**).


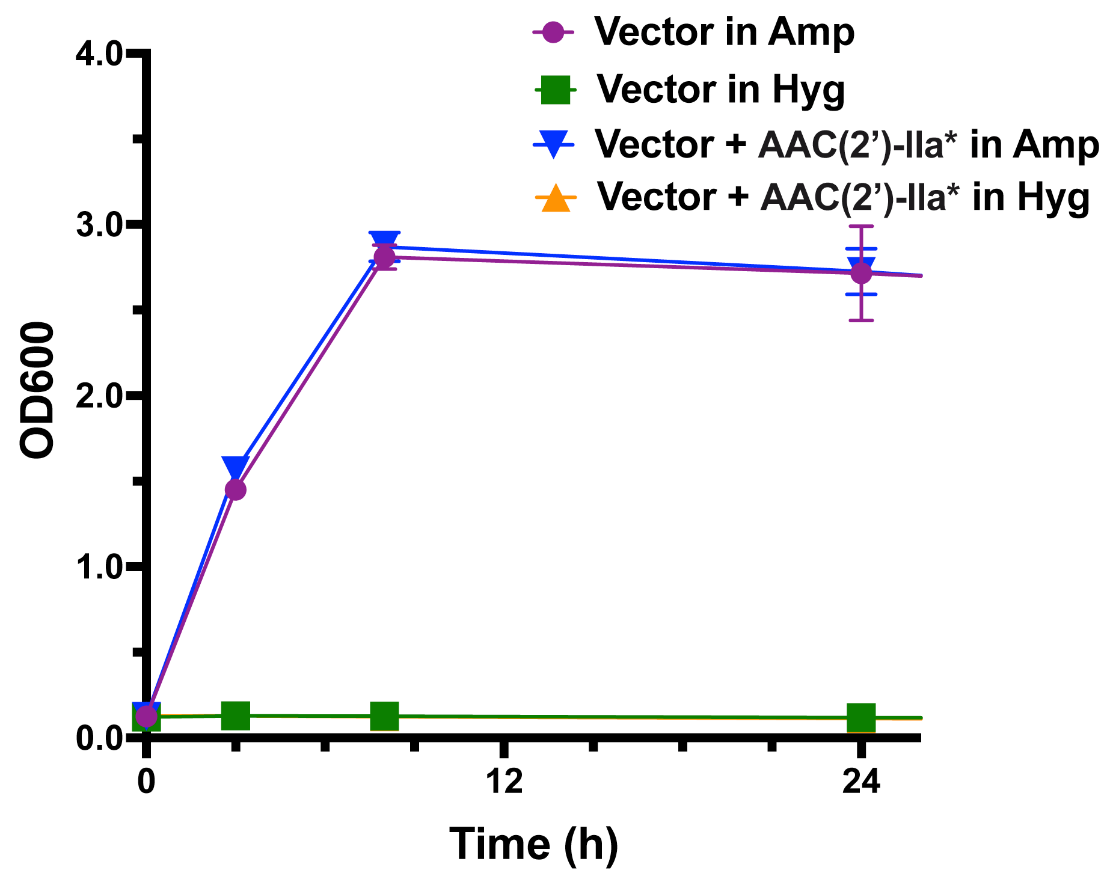


**Supplementary Figure 5.** *E. coli* TOP10 harboring the pEX18.AP plasmid with an uncharacterized gene (AAC(2’)-lla*) with 72.57% identity to a known AAC(2’)-IIa aminoglycoside resistance gene cloned from pSDM007 was grown in LB media with and without hygromycin B. OD_600_ values are shown of TOP10pEX18.AP in 100 µg/mL ampicillin (purple circle), TOP10pEX18.AP in 100 µg/mL hygromycin B (green square), TOP10pEX18.AP_AAC(2’)-IIa* in 100 µg/mL ampicillin (blue triangle), and TOP10pEX18.AP_AAC(2’)-IIa* in 100 µg/mL hygromycin B (yellow triangle). Error bars indicate standard error from two independent experiments (n=2).

**Supplementary Table 1.** Average nucleotide identity and aligned nucleotide percentage (shown in brackets) of all the strains closely related to *P. hygromyciniae sp. nov. strain SDM007^T^*

| **10** | * | 90.15  [73.07] | 97.47  [84.33] | 90.24  [71.93] | 90.13  [70.90] | 90.18  [71.18] | 90.07  [70.67] | 90.43  [71.67] | 90.32  [71.60] | 90.36  [69.60] |
| --- | --- | --- | --- | --- | --- | --- | --- | --- | --- | --- |
| **9** | 90.36  [77.03] | * | 90.18  [74.50] | 98.14  [86.88] | 90.47  [75.22] | 91.00  [74.83] | 97.71  [83.47] | 90.99  [75.46] | 91.05  [74.75] | 98.20  [85.52] |
| **8** | 97.61  [87.60] | 90.19  [72.83] | * | 90.43  [72.82] | 90.19  [73.78] | 90.57  [74.22] | 90.35  [72.86] | 90.86  [74.50] | 90.79  [74.09] | 90.52  [71.66] |
| **7** | 90.34  [77.63] | 98.08  [89.50] | 90.37  [76.29] | * | 90.46  [75.42] | 90.98  [78.50] | 98.22  [89.52] | 91.13  [77.44] | 91.08  [77.94] | 98.51  [89.45] |
| **6** | 90.02  [76.47] | 90.36  [75.53] | 90.43  [77.43] | 90.60  [73.76] | * | 89.89  [73.21] | 90.62  [73.58] | 90.15  [74.13] | 90.02  [73.02] | 90.86  [73.04] |
| **5** | 90.32  [79.48] | 90.91  [79.68] | 90.56  [79.78] | 90.95  [80.62] | 89.85  [76.69] | * | 91.02  [80.99] | 99.18  [91.24] | 99.07  [92.45] | 91.09  [80.20] |
| **4** | 90.12  [76.90] | 97.65  [86.31] | 90.29  [76.08] | 98.29  [89.58] | 90.49  [75.37] | 90.92  [78.80] | * | 91.03  [78.06] | 91.10  [78.93] | 98.01  [88.08] |
| **3** | 90.53  [80.02] | 91.06  [79.83] | 90.88  [80.04] | 91.21  [79.89] | 90.07  [78.11] | 99.25  [91.83] | 91.21  [79.82] | * | 99.24  [92.65] | 91.25  [80.18] |
| **2** | 90.59  [80.38] | 91.19  [79.59] | 90.90  [79.89] | 91.23  [80.85] | 90.06  [77.19] | 99.17  [93.06] | 91.22  [81.53] | 99.26  [92.79] | * | 91.24  [81.09] |
| **1** | 90.48  [79.36] | 98.35  [92.35] | 90.47  [78.72] | 98.69  [93.92] | 90.76  [78.41] | 91.21  [81.72] | 98.18  [92.05] | 91.27  [81.63] | 91.22  [82.26] | * |
| **ANIb [aligned nucleotides]** | *Pseudomonas sp* ICMP8385 | *P. proteolytica* BS2985 | *P. gessardii* DSM17152 | *Pseudomonas sp*. LG1D9 | *P. fluorescens* NCTC10392 | *Pseudomonas sp* 25R14 | *P. fluorescens* ATCC17400 | *Pseudomonas sp* FH4 | *P. brenneri* BS2771 | *P. brenneri* BIGb0273 |

1. *P. brenneri* BIGb0273, 2. *P. brenneri* BS2771, 3. *Pseudomonas sp.* FH, 4. *P. fluorescens* ATCC17400, 5. *Pseudomonas sp.* 25R14, 6. *P. fluorescens* NCTC10392, 7. *Pseudomonas sp.* LG1D9, 8. *P. gessardii* DSM17152, 9. *P. proteolytica* BS2985, 10. *Pseudomonas sp* ICMP8385

**Supplementary Table 2.** Growth of *P. hygromyciniae sp. nov. strain SDM007^T^* on carbon sources. SDM007^T^ was inoculated into each carbon source using phenotypic microarrays. Growth was measured after 12 hours. Green rows highlight carbon sources that supported growth and red rows highlight caron sources that did not support growth based on the minimum OD_410_ value of 0.200. N = “None or low growth”; Y = “Yes, growth”

| **Carbon source** | **OD_410_ value** | **Standard error** | **Growth?** |
| --- | --- | --- | --- |
| 1,2-Propanediol | 0.076 | 0.031 | N |
| 2,3-Butanediol | 0.071 | 0.041 | N |
| 2,3-Butanedione | 0.017 | 0.017 | N |
| 2-Aminoethanol | 0.296 | 0.038 | Y |
| 2-Deoxy adenosine | 0.043 | 0.043 | N |
| 2-Deoxy-D-Ribose | 0.040 | 0.040 | N |
| 2-Hydroxy benoic acid | 0.008 | 0.008 | N |
| 3-0-β-D-Galactopyranosyl-D-arabinose | 0.057 | 0.033 | N |
| 3-Hydroxy-2-butanone | 0.017 | 0.017 | N |
| 3-Methyl glucose | 0.036 | 0.036 | N |
| 4-Hydroxy benzoic acid | 0.391 | 0.082 | Y |
| 5-Keto-D-gluconic acid | 0.101 | 0.101 | N |
| Acetamide | 0.012 | 0.012 | N |
| Acetic acid | 0.373 | 0.064 | Y |
| Acetoacetic acid | 0.090 | 0.049 | N |
| Adenosine | 0.305 | 0.024 | Y |
| Adonitol | 0.091 | 0.027 | N |
| Amygdalin | 0.020 | 0.020 | N |
| Arbutin | 0.003 | 0.003 | N |
| Bromo succinic acid | 0.472 | 0.062 | Y |
| Butyric acid | 0.012 | 0.012 | N |
| Capric acid | 0.414 | 0.048 | Y |
| Caproic acid | 0.168 | 0.168 | N |
| Chondroitin sulfate C | 0.107 | 0.031 | N |
| Citraconic acid | 0.041 | 0.041 | N |
| Citramalic acid | 0.022 | 0.022 | N |
| Citric acid | 1.053 | 0.001 | Y |
| D,L-Carnitine | 0.376 | 0.013 | Y |
| D,L-Malic acid | 0.686 | 0.030 | Y |
| D,L-Octopamine | 0.020 | 0.020 | N |
| D,L-α-Glycerol-phosphate | 0.427 | 0.002 | Y |
| D-Alanine | 0.743 | 0.039 | Y |
| D-Arabinose | 0.046 | 0.046 | N |
| D-Arabitol | 0.283 | 0.055 | Y |
| D-Aspartic acid | 0.021 | 0.021 | N |
| D-Cellobiose | 0.053 | 0.053 | N |
| Dextrin | 0.059 | 0.043 | N |
| D-Fructose | 0.587 | 0.013 | Y |
| D-Fructose-6-phosphate | 0.056 | 0.042 | N |
| D-Fucose | 0.032 | 0.032 | N |
| D-Galactonic acid-γ-lactone | 0.985 | 0.037 | Y |
| D-Galactose | 0.609 | 0.085 | Y |
| D-Galacturonic acid | 0.035 | 0.035 | N |
| D-Gluconic acid | 1.032 | 0.045 | Y |
| D-Glucosamine | 0.293 | 0.039 | Y |
| D-Glucosaminic acid | 0.077 | 0.040 | N |
| D-Glucose-1-phosphate | 0.028 | 0.028 | N |
| D-Glucose-6-phosphate | 0.010 | 0.010 | N |
| D-Glucuronic acid | 0.021 | 0.021 | N |
| Dihydroxyacetone | 0.423 | 0.040 | Y |
| D-Lactic acid methyl ester | 0.048 | 0.048 | N |
| D-Malic acid | 0.199 | 0.092 | N |
| D-Mannitol | 1.150 | 0.052 | Y |
| D-Mannose | 0.916 | 0.048 | Y |
| D-Melezitose | 0.052 | 0.052 | N |
| D-Melibiose | 0.038 | 0.038 | N |
| D-Psicose | 0.089 | 0.086 | N |
| D-Raffinose | 0.012 | 0.012 | N |
| D-Ribono-1,4-lactone | 1.375 | 0.014 | Y |
| D-Ribose | 0.357 | 0.028 | Y |
| D-Saccharic acid | 0.013 | 0.013 | N |
| D-Serine | 0.930 | 0.001 | Y |
| D-Sorbitol | 0.028 | 0.028 | N |
| D-Tagatose | 0.052 | 0.019 | N |
| D-Tartaric acid | 0.032 | 0.032 | N |
| D-Threonine | 0.048 | 0.048 | N |
| D-Trehalose | 1.043 | 0.040 | Y |
| Dulcitol | 0.032 | 0.032 | N |
| D-Xylose | 0.028 | 0.028 | N |
| Formic acid | 0.077 | 0.036 | N |
| Fumaric acid | 1.033 | 0.026 | Y |
| Gelatin | 0.032 | 0.027 | N |
| Gentiobiose | 0.006 | 0.006 | N |
| Glucuronamide | 0.025 | 0.025 | N |
| Glycerol | 0.702 | 0.069 | Y |
| Glycine | 0.123 | 0.042 | N |
| Glycogen | 0.036 | 0.036 | N |
| Glycolic acid | 0.041 | 0.041 | N |
| Glycolic acid | 0.022 | 0.022 | N |
| Glycyl-L-aspartic acid | 0.034 | 0.034 | N |
| Glycyl-L-glutamic acid | 0.296 | 0.050 | Y |
| Glycyl-L-proline | 0.031 | 0.031 | N |
| Glyoxylic acid | 0.072 | 0.069 | N |
| Hydroxy-L-proline | 1.647 | 0.113 | Y |
| i-Erythritol | 0.254 | 0.009 | Y |
| Inosine | 0.612 | 0.013 | Y |
| Inulin | 0.247 | 0.091 | Y |
| Itaconic acid | 0.000 | 0.000 | N |
| Lactitol | 0.033 | 0.033 | N |
| Lactulose | 0.044 | 0.044 | N |
| L-Alaninamide | 0.909 | 0.022 | Y |
| L-Alanine | 0.751 | 0.027 | Y |
| L-Alanyl-glycine | 0.666 | 0.039 | Y |
| Laminarin | 0.075 | 0.055 | N |
| L-Arabinose | 0.018 | 0.018 | N |
| L-Arabitol | 0.000 | 0.000 | N |
| L-Arginine | 0.509 | 0.060 | Y |
| L-Asparagine | 1.122 | 0.042 | Y |
| L-Aspartic acid | 0.992 | 0.079 | Y |
| L-Fucose | 0.020 | 0.020 | N |
| L-Galactonic acid-γ-lactone | 0.034 | 0.034 | N |
| L-Glucose | 0.013 | 0.013 | N |
| L-Glutamic acid | 0.880 | 0.030 | Y |
| L-Glutamine | 1.068 | 0.054 | Y |
| L-Histidine | 1.127 | 0.055 | Y |
| L-Homoserine | 0.000 | 0.000 | N |
| L-Isoleucine | 1.347 | 0.108 | Y |
| L-Lactic acid | 0.868 | 0.033 | Y |
| L-Leucine | 1.209 | 0.031 | Y |
| L-Lysine | 0.113 | 0.036 | N |
| L-Lyxose | 0.043 | 0.043 | N |
| L-Malic acid | 0.796 | 0.098 | Y |
| L-Methionine | 0.064 | 0.027 | N |
| L-Ornithine | 0.838 | 0.060 | Y |
| L-Phenylalanine | 0.512 | 0.065 | Y |
| L-Proline | 1.007 | 0.055 | Y |
| L-Pyroglutamic acid | 0.769 | 0.035 | Y |
| L-Rhamnose | 0.029 | 0.029 | N |
| L-Serine | 0.888 | 0.070 | Y |
| L-Sorbose | 0.039 | 0.039 | N |
| L-Tartaric acid | 0.045 | 0.023 | N |
| L-Threonine | 0.173 | 0.059 | N |
| L-Valine | 0.347 | 0.017 | Y |
| Malonic acid | 0.450 | 0.006 | Y |
| Maltitol | 0.034 | 0.034 | N |
| Maltose | 0.078 | 0.054 | N |
| Maltotriose | 0.122 | 0.096 | N |
| Mannan | 0.025 | 0.025 | N |
| Melibionic acid | 0.046 | 0.039 | N |
| Methyl pyruvate | 0.559 | 0.040 | Y |
| m-Hydroxy phenyl acetic acid | 0.032 | 0.032 | N |
| Mono methyl succinate | 0.091 | 0.056 | N |
| m-Tartaric acid | 0.025 | 0.025 | N |
| Mucic acid | 1.510 | 0.143 | Y |
| Myo-inositol | 0.593 | 0.013 | Y |
| N-Acetyl-D-galactosamine | 0.030 | 0.028 | N |
| N-Acetyl-D-glucosamine | 0.566 | 0.060 | Y |
| N-Acetyl-D-glucosaminitol | 0.062 | 0.036 | N |
| N-Acetyl-L-glutamic acid | 1.027 | 0.035 | Y |
| N-Acetyl-Neuraminic acid | 0.009 | 0.009 | N |
| N-Acetyl-β-D-mannosamine | 0.110 | 0.024 | N |
| Negative Control | 0.000 | 0.000 | N |
| Oxalic acid | 0.033 | 0.033 | N |
| Oxalomalic acid | 0.045 | 0.038 | N |
| Palatinose | 0.035 | 0.035 | N |
| Pectin | 0.085 | 0.074 | N |
| Phenylethyl-amine | 0.039 | 0.039 | N |
| p-Hydroxy phenyl acetic acid | 0.650 | 0.049 | Y |
| Propionic acid | 0.607 | 0.011 | Y |
| Putrescine | 0.500 | 0.108 | Y |
| Pyruvic acid | 0.711 | 0.002 | Y |
| Quinic acid | 0.935 | 0.114 | Y |
| Salicin | 0.024 | 0.024 | N |
| Sebacic acid | 0.777 | 0.048 | Y |
| Sec-Butylamine | 0.000 | 0.000 | N |
| Sedoheptulosan | 0.037 | 0.037 | N |
| Sorbic acid | 0.035 | 0.020 | N |
| Stachyose | 0.029 | 0.025 | N |
| Succinamic acid | 1.253 | 0.023 | Y |
| Succinic acid | 0.863 | 0.050 | Y |
| Sucrose | 0.033 | 0.033 | N |
| Thymidine | 0.054 | 0.054 | N |
| Tricarballylic acid | 0.028 | 0.028 | N |
| Turanose | 0.044 | 0.035 | N |
| Tween 20 | 0.529 | 0.055 | Y |
| Tween 40 | 0.428 | 0.043 | Y |
| Tween 80 | 0.384 | 0.042 | Y |
| Tyramine | 0.763 | 0.038 | Y |
| Uridine | 0.302 | 0.042 | Y |
| Xylitol | 0.030 | 0.030 | N |
| α-Cyclodextrin | 0.021 | 0.013 | N |
| α-D-Glucose | 1.227 | 0.025 | Y |
| α-D-Lactose | 0.051 | 0.025 | N |
| α-Hydroxy butyric acid | 0.148 | 0.031 | N |
| α-Hydroxy glutaric acid-γ-lactone | 0.655 | 0.061 | Y |
| α-Keto-butyric acid | 0.153 | 0.055 | N |
| α-Keto-glutaric acid | 0.806 | 0.026 | Y |
| α-Keto-valeric acid | 0.066 | 0.066 | N |
| α-Methyl-D-galactoside | 0.068 | 0.047 | N |
| α-Methyl-D-glucoside | 0.040 | 0.022 | N |
| α-Methyl-D-mannoside | 0.055 | 0.055 | N |
| β-Cyclodextrin | 0.031 | 0.031 | N |
| β-D-Allose | 0.005 | 0.005 | N |
| β-Hydroxy butyric acid | 1.142 | 0.091 | Y |
| β-Methyl-D-galactoside | 0.040 | 0.009 | N |
| β-Methyl-D-glucoside | 0.077 | 0.015 | N |
| β-Methyl-D-glucuronic acid | 0.047 | 0.034 | N |
| β-Methyl-D-xyloside | 0.016 | 0.016 | N |
| γ-Amino butyric acid | 1.670 | 0.021 | Y |
| γ-Cyclodextrin | 0.007 | 0.007 | N |
| δ-Amino valeric acid | 0.199 | 0.005 | N |

**Supplementary Table 3.** Putative conjugation genes on plasmid pSDM007. Query coverage, percent identity, and highest similarity were generated using BLAST (blastn) against the NCBI Gammaproteobacteria database. Location (bp) is based on accession number NZ_CP070507 sequence.

| **pSDM007 genes predicted to play a role in conjugation** | | | | |
| --- | --- | --- | --- | --- |
| **Gene or predicted function** | **Location (bp)** | **Query Cover (%)** | **Identity (%)** | **Highest Similarity** |
| *traG* | 131,019-132,658 | 100 | 84.27 | *P. putida* plasmid pDK1 |
| Pilus assembly | 132,670-134,073 | 99 | 84.96 | *P. rhodesiae* strain BS2777 |
| Pilus assembly | 134,070-135,095 | 100 | 85.38 | *P. aeruginosa* plasmid p1160-VIM |
| DNA primase | 136,378-137,001 | 100 | 88.47 | *P. putida* plasmid pDK1 |
| Nuclease-related domain-containing protein | 138,615-139,334 | 100 | 84.03 | *P.* *putida* plasmid pDK1 |
| *traU* | 139,384-140,385 | 100 | 86.27 | *P. rhodesiae* strain BS2777 |
| Conjugal transfer protein | 140,420-141,661 | 100 | 82.13 | *P. aeruginosa* plasmid p1160-VIM |
| Pilus assembly protein | 141,655-142,143 | 99 | 85.66 | *P. aeruginosa* plasmid p1160-VIM |
| *traC* DNA transfer | 142,227-144,692 | 100 | 86.54 | *P.* *aeruginosa* plasmid p1160-VIM |
| *traV* | 152,800-153,225 | 100 | 83.57 | *P.* *poae* strain CAP-2018 |
| Pilus assembly protein | 153,225-154,546 | 99 | 80.06 | *P. resinovorans* plasmid pCAR1.3 |
| Conjugal transfer protein | 154,565-155,604 | 100 | 80.58 | *P. aeruginosa* plasmid p1160-VIM |
| *traE* | 155,782-156,423 | 100 | 81.93 | *P.* *aeruginosa* plasmid p1160-VIM |
| *traL* | 145,423-156,710 | 100 | 86.46 | *P. aeruginosa* plasmid p1160-VIM |
| Conjugal transfer protein | 158,590-160,419 | 100 | 76.08 | *P. veronii* strain 1YdBTEX2 |
| Relaxase | 160,416-162,969 | 100 | 82.28 | *P.* *veronii* strain 1YdBTEX2 |
| *traN* mating pair stabilization | 174,658-175,913 | 100 | 74.78 | *P.* *aeruginosa* plasmid pNK546b |

**Supplementary Table 4.** Strains used in this study

| **Species** | **Strain** | **Notes** | **Reference** |
| --- | --- | --- | --- |
| *E. coli* | TOP10 | Chemical-competent | Invitrogen |
| *E. coli* | TOP10pEX18.AP | Ampicillin resistant | This study |
| *E. coli* | TOP10pEX18.AP_hph | Ampicillin resistant and harboring SDM007^T^ *hph* gene | This study |
| *E. coli* | TOP10pEX18.AP_ AAC(2’)-IIa* | Ampicillin resistant and harboring SDM007^T^ gene of unknown function with 72.57% homology to a AAC(2’)-IIa resistance gene | This study |
| *P. aeruginosa* | PAO1 | Canonical *P. aeruginosa* isolate |  |
| *P. fluorescens* | ATCC 17569 | Strain 202 [PJ 372] | ATCC |
| *P. hygromyciniae sp. nov.* | SDM007^T^ | Isolated from hygromycin B stock | This study |
| *P. hygromyciniae sp. nov.* | SDM007_2 | Isolated from 2^nd^ bottle of hygromycin B stock | This study |

**Supplementary Table 5.** Plasmids used in this study.

| **Plasmid** | **Notes** | **Reference** |
| --- | --- | --- |
| pEX18.AP | Ampicillin resistance, *oriT*, *sacB*, *lacZα*, MCS from pUC18 | (1) |

**Supplementary Table 6.** Primers used in this study.

| Primer | Notes | Sequence (5’-3’) |
| --- | --- | --- |
| TT201 | Forward primer for cloning SDM007^T^ gene with 72.57% identity to AAC(2’)-IIa; EcoRI cut adapter | gatccccgggtaccgagctcgCGCGGTCACCTGATCCATTTC |
| TT202 | Reverse primer for cloning SDM007^T^ gene with 72.57% identity to AAC(2’)-IIa; EcoRI cut adapter | gctatgaccatgattacgCCGGCGACTGCATTCTTGATG |
| TT203 | Forward primer for cloning SDM007^T^ *hph* gene; EcoRI cut adapter | gatccccgggtaccgagctcgGCGAGTGACGCATCGGATCTC |
| TT204 | Reverse primer for cloning SDM007^T^ *hph* gene; EcoRI cut adapter | gctatgaccatgattacgGACCAAACGATGGGAGGCTGC |
| pEX18_F | Forward pEX18 multiple cloning site confirmation primer | ACTTCTGAGTTCGGCATGGGG |
| pEX18_R | Reverse pEX18 multiple cloning site confirmation primer | GAAAGCGGGCAGTGAGCGCA |

1. Hoang TT, Karkhoff-Schweizer RR, Kutchma AJ, Schweizer HP. 1998. A broad-host-range Flp-FRT recombination system for site-specific excision of chromosomally-located DNA sequences: application for isolation of unmarked *Pseudomonas aeruginosa* mutants. Gene 212:77-86.
